# INDIVIDUAL AND DYADIC PREDICTORS OF TECHNOLOGY ADOPTION: IMPLICATIONS FOR HEALTH AND WELL-BEING

**DOI:** 10.1093/geroni/igz038.052

**Published:** 2019-11-08

**Authors:** William J Chopik, Shelia R Cotten

**Affiliations:** Michigan State University, East Lansing, Michigan, United States

## Abstract

Technology has the ability to enhance and enrich the lives of older adults by facilitating better relationships, health, and well-being. However, older adults vary in how often—and even whether—they use information and communication technologies (ICTs). Further, interactions and relationships with people in their immediate social networks might have implications for whether or not older adults adopt ICTs. In two studies of individuals (N=595 participants; Mage=67.09; 56% Female; 69.2% White) and couples (N=542 couples; Mage=63.65; 50% Female; 83.9% White), I examined individual and dyadic predictors of technology adoption among older adults. Among a wide array of individual difference constructs, the most reliable predictor of technology adoption in both individuals and their spouses was need for cognition (.08 ≤ r ≤ .23). The results will be discussed in the context of how individual differences modulate adoption and the benefits accrued from ICTs across the lifespan.

